# Supplementary material for: Revisiting the Self-Confidence and Sport Performance Relationship: A Systematic Review with Meta-Analysis
Source: Int J Environ Res Public Health. 2022 May 24;19(11):6381. doi: 10.3390/ijerph19116381 (PMC9180271; doi:10.3390/ijerph19116381)
Supplement: Supplementary file 1 [file ijerph-19-06381-s001.zip › ijerph-1695066-supplementary.pdf]

Supplement 1. Search strategy

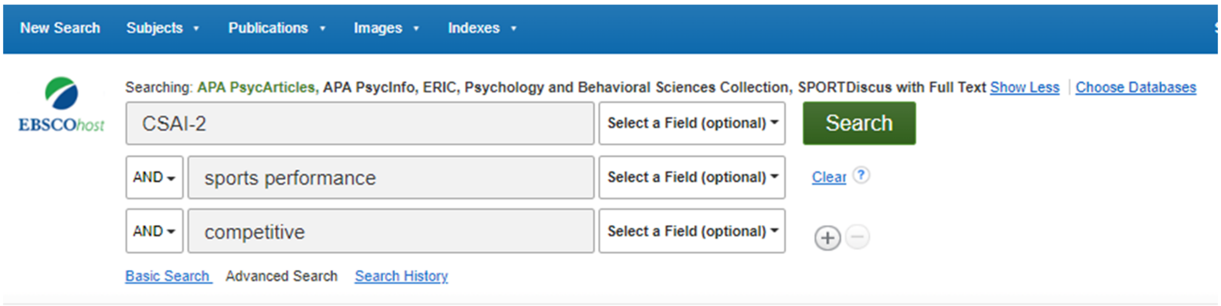

1. EBSCO
2. APA PsycArticles, APA PsycInfo
3. Advanced ERIC, Psychology and Behavioral Sciences Collection, SPORTDiscus
4. Typed key terms
5. CSAI-2 Box 1
6. Sport performance Box 2
7. Competitive Box 3
8. Limit to: scholarly peer-reviewed journals
9. Page options: 50
10. Limit time: 2002-2020 [computer changed to 2003]
11. Started new search focused on Trait Sport Confidence Inventory
12. Same EBSCO databases
13. TSCI Box 1
14. Sport performance Box 2
15. Competitive Box 3
16. Limit to: scholarly peer-reviewed journals
17. 2 results from 1991-2001
18. Trait-Sport Confidence Inventory Box 1
19. Page options: 50
20. 65 results from 1985-2021
21. Changed to 2016-2021
22. 29 results
23. Confidence Box 1
24. Sport performance Box 2
25. Competitive Box 3
26. 313 results

27. Limit to: scholarly peer-reviewed journals
28. Page options: 50
29. Changed to 2020-2021
30. 29 results
31. Changed to 2018-2019
32. 37 results
33. Confidence Box 1
34. Sport performance Box 2
35. Competitive Box 3
36. Change to 2016-2017
37. Limit to: scholarly peer-reviewed journals
38. Page options: 50
39. 31 results
40. Change to 2014-2015
41. 51 results
42. 2012-2013
43. 24 results
44. 2010-2011
45. 16 results
46. 2008-2009
47. 11 results
48. 2006-2007
49. 8 results
50. 1983-1989
51. 5 results
52. 1990-1992
53. 7 results
54. 1993-1995
55. 9 results
56. 1996-1998
57. 20 results
58. 1999-2000
59. 7 results
60. 2001-2003

61. 8 results
62. 2004-2005
63. 19 results

Supplement 2. Risk of individual study bias ratings

Study	Sample	Random selection	Nonresponse	Direct collection	Confidence measure	Sport measure	Data collection	Anonymity	Time	Total Score	Grouping
Armes Alejo [37]	3	1	3	3	2	3	3	2	1	21	M
Barnes et al. [38]	3	1	3	3	2	3	3	3	2	23	H
Beattie et al. [39]	3	1	3	3	3	3	3	1	2	22	H
Bejek & Hagtvet [8]	3	1	3	3	3	3	3	1	2	22	H
Bejek & Hagtvet [8]	2	1	3	3	3	3	3	1	2	21	M
Bird & Horn [40]	2	1	2	3	3	1	3	2	2	19	L
Bueno et al. [41]	2	1	1	2	2	1	3	2	1	15	L
Burton [42]	3	1	3	3	2	3	3	1	2	21	M
Burton [42]	3	1	3	3	2	3	3	1	2	21	M
Chapman et al. [43]	2	1	3	3	3	3	3	1	2	21	M
Cox et al. [44]	1	1	3	3	3	2	3	3	3	22	H
Draper et al. [3]	2	1	3	3	2	3	3	2	3	22	H
Edwards & Hardy [45]	2	1	3	3	2	1	3	3	2	20	L
Gayton & Nickless [46]	2	1	1	3	2	3	3	1	3	19	L
Hassmén et al. [4]	3	1	3	3	2	3	3	2	2	22	H
Heazlewood & Burke [47]	2	1	3	3	2	3	3	1	1	19	L
Iso-Ahola & Blanchard [48]	2	1	3	3	1	3	3	1	3	20	L
Jerome & Williams [9]	2	1	3	3	2	2	3	3	2	21	M
Jones et al. [49]	1	1	3	3	2	1	3	3	3	20	L
Kais et al. [50]	2	1	3	3	3	3	3	3	1	22	H
Kais et al. [10]	2	1	3	3	3	1	3	3	1	20	L
Laborde et al. [51]	1	1	3	3	3	2	3	2	3	21	M
Lautenbach et al. [52]	1	1	3	3	2	2	3	2	3	20	L
Levy et al. [53]	1	1	3	3	3	1	3	2	2	19	L
Martin et al. [54]	2	1	3	3	2	3	3	2	2	21	M
Maynard & Howe [55]	2	1	3	3	2	1	3	1	2	18	L
McAuley [7]	3	1	3	3	2	3	3	3	3	24	H
McCann et al. [56]	3	1	3	3	2	2	3	2	3	22	H

Nicholls et al. [57]	2	1	3	3	3	1	3	2	1	19	L
Perreault & Marisi [58]	3	1	2	3	3	2	3	1	2	20	L
Pessoa da Costa et al. [59]	2	1	3	3	2	2	3	2	2	20	L
Pinto & Vázquez [60]	2	1	3	3	3	3	3	2	1	21	M
Psychountaki & Zervas [61]	1	1	3	3	2	1	3	1	2	17	L
Rodrigo et al. [62]	2	1	3	3	3	1	3	1	2	19	L
Sanchez et al. [63]	3	1	3	3	3	3	3	2	2	23	H
Swain & Jones [64]	3	1	3	3	3	3	3	1	2	22	H
Terry & Slade [5]	2	1	3	3	2	3	3	3	2	22	H
Terry & Youngs [65]	2	1	3	3	2	1	3	3	2	20	L
Terry et al. [21]	3	1	3	3	2	3	3	3	2	23	H
Totterdell [66]	3	1	2	3	1	2	3	1	3	19	L
Tsopani et al. [67]	1	1	2	3	2	3	3	1	2	18	L
Vadocz et al. [68]	3	1	3	3	3	3	3	2	1	22	H
Zienius et al. [6]	2	1	3	3	2	3	3	2	3	22	H
Mean	2.19	1.00	2.81	2.97	2.35	2.30	3.00	1.88	2.06	20.58	
SD	0.70	0.00	0.50	0.15	0.57	0.86	0.00	0.79	0.67	1.76	

Number abbreviations: 3 = low risk of bias, 2 = medium risk of bias, 1 = high risk of bias; Letter abbreviations: H = higher quality study, M = medium quality study, L = low quality study.

Supplement 3. Comparison of Results

**Analysis**	**Current Meta**	**Craft et al. [xx]**	**Woodman and Hardy [xx]**	**Summary**
	k, r [95% CI]	k, r [95% CI]	k, r [95% CI]	
Overall relationship	47, 0.25 [0.19, 0.31]	29, 0.25 [0.20, 0.28]	36, 0.27 [0.20, 0.33]	Replicated
Terry's sport propositions				
Individual sport	31, 0.29 [0.22, 0.35]	17, 0.49 [0.42, 0.56]*	?, 0.25 [0.16, 0.34]	
Team sport	14, 0.14 [0.03, 0.25]	38, 0.19 [0.10, 0.29]*	?, 0.19 [0.05, 0.34]	Congruent with Woodman and Hardy [xx]
Closed skill	24, 0.28 [0.19, 0.36]	34, 0.09 [0.01, 0.18]*	Not analyzed	
Open skill	21, 0.21 [0.12, 0.30]	21, 0.55 [0.49, 0.61]*	Not analyzed	Incongruent
Time of Confidence Administration				
<15 (15 min or less)	13, 0.25 [0.12, 0.37]	18, 0.20 [0.10, 0.30]*	Not analyzed	
16-30	9, 0.19 [0.02, 0.34]	8, 0.44 [0.26, 0.61]*	Not analyzed	
31-59 (60)	19, 0.25 [0.16, 0.34]	11, 1.07 [0.98, 1.16]*	Not analyzed	
>1 (1-4 hrs.)	6, 0.30 [0.15, 0.44]	10, 0.16 [0.06, 0.25]*	Not analyzed	Incongruent for the most, congruent only with >15 min.
Individual differences				
Male	43, percent male meta-regression $R^2 = 0.24$ ($r = 0.49$)	Not analyzed	?, 0.29 [0.18, 0.40]	

Female		Not analyzed	?, 0.04 [-0.05, 0.13]	Congruent, confidence-performance relationship greater, more important for males than females
High standard	22, 0.20 [0.14, 0.27]	Not analyzed	?, 0.33 [0.19, 0.47]	
Low standard	24, 0.26 [0.18, 0.34]	Not analyzed	?, 0.16 [0.06, 0.25]	Fairly consistent, I placed elite and college in the high standard group; all club, high school and recreational in the low standard group
Elite	14, 0.22 [0.10, 0.32]	17, 0.10 [0.01, 0.21]	Not analyzed	
College	8, 0.21 [0.08, 0.34]	16, 0.12 [0.01, 0.24]	Not analyzed	
European club	6, 0.20 [0.04, 0.34]	11, 1.24 [1.14, 1.33]	Not analyzed	
College PE students	Not in our inclusion criteria.	9, 0.23 [0.09, 0.36]		European club way off, the rest are small effects for us and Craft et al.

*Craft et al. [xx] values slope coefficients.

Supplement 4. PRISMA Checklist

Section and Topic	Item #	Checklist item	Location where item is reported
**TITLE**			
Title	1	Identify the report as a systematic review.	Yes
**ABSTRACT**			
Abstract	2	See the PRISMA 2020 for Abstracts checklist.	Abstract written with MDPI 200 word limit in mind.
**INTRODUCTION**			
Rationale	3	Describe the rationale for the review in the context of existing knowledge.	Introduction
Objectives	4	Provide an explicit statement of the objective(s) or question(s) the review addresses.	Section 1.1. Research questions
**METHODS**			
Eligibility criteria	5	Specify the inclusion and exclusion criteria for the review and how studies were grouped for the syntheses.	Section 2.1
Information sources	6	Specify all databases, registers, websites, organisations, reference lists and other sources searched or consulted to identify studies. Specify the date when each source was last searched or consulted.	Section 2.2
Search strategy	7	Present the full search strategies for all databases, registers and websites, including any filters and limits used.	Section 2.2
Selection process	8	Specify the methods used to decide whether a study met the inclusion criteria of the review, including how many reviewers screened each record and each report retrieved, whether they worked independently, and if applicable, details of automation tools used in the process.	Section 2.3
Data collection process	9	Specify the methods used to collect data from reports, including how many reviewers collected data from each report, whether they worked independently, any processes for obtaining or confirming data from study investigators, and if applicable, details of automation tools used in the process.	Section 2.4
Data items	10a	List and define all outcomes for which data were sought. Specify whether all results that were compatible with each outcome domain in each study were sought (e.g. for all measures, time points, analyses), and if not, the methods used to decide which results to collect.	Section 2.4
	10b	List and define all other variables for which data were sought (e.g. participant and intervention characteristics, funding sources). Describe any assumptions made about any missing or unclear information.	Section 2.4
Study risk of bias assessment	11	Specify the methods used to assess risk of bias in the included studies, including details of the tool(s) used, how many reviewers assessed each study and whether they worked independently, and if applicable, details of automation tools used in the process.	Section 2.5, 2.7
Effect measures	12	Specify for each outcome the effect measure(s) (e.g. risk ratio, mean difference) used in the synthesis or presentation of results.	Section 2.6
Synthesis methods	13a	Describe the processes used to decide which studies were eligible for each synthesis (e.g. tabulating the study intervention characteristics and comparing against the planned groups for each synthesis (item #5)).	Section 2.6
	13b	Describe any methods required to prepare the data for presentation or synthesis, such as handling of missing summary	Section 2.6

Section and Topic	Item #	Checklist item	Location where item is reported
		statistics, or data conversions.	
	13c	Describe any methods used to tabulate or visually display results of individual studies and syntheses.	Section 2.6
	13d	Describe any methods used to synthesize results and provide a rationale for the choice(s). If meta-analysis was performed, describe the model(s), method(s) to identify the presence and extent of statistical heterogeneity, and software package(s) used.	Section 2.6
	13e	Describe any methods used to explore possible causes of heterogeneity among study results (e.g. subgroup analysis, meta-regression).	Section 2.6
	13f	Describe any sensitivity analyses conducted to assess robustness of the synthesized results.	Section 2.7
Reporting bias assessment	14	Describe any methods used to assess risk of bias due to missing results in a synthesis (arising from reporting biases).	Study level reliability, Section 2.5
Certainty assessment	15	Describe any methods used to assess certainty (or confidence) in the body of evidence for an outcome.	Found in full in section 3.5, and table 5
**RESULTS**			
Study selection	16a	Describe the results of the search and selection process, from the number of records identified in the search to the number of studies included in the review, ideally using a flow diagram.	Section 3.1
	16b	Cite studies that might appear to meet the inclusion criteria, but which were excluded, and explain why they were excluded.	Mentioned an example line 169-170, Excel sheet of other examples available from first author
Study characteristics	17	Cite each included study and present its characteristics.	Section 3.1
Risk of bias in studies	18	Present assessments of risk of bias for each included study.	Section 3.2
Results of individual studies	19	For all outcomes, present, for each study: (a) summary statistics for each group (where appropriate) and (b) an effect estimate and its precision (e.g. confidence/credible interval), ideally using structured tables or plots.	Section 3.3, Fig 2
Results of syntheses	20a	For each synthesis, briefly summarise the characteristics and risk of bias among contributing studies.	Section 3.3
	20b	Present results of all statistical syntheses conducted. If meta-analysis was done, present for each the summary estimate and its precision (e.g. confidence/credible interval) and measures of statistical heterogeneity. If comparing groups, describe the direction of the effect.	Section 3.3, 3.4
	20c	Present results of all investigations of possible causes of heterogeneity among study results.	Section 3.3
	20d	Present results of all sensitivity analyses conducted to assess the robustness of the synthesized results.	Fig 3
Reporting biases	21	Present assessments of risk of bias due to missing results (arising from reporting biases) for each synthesis assessed.	Section 3.3
Certainty of	22	Present assessments of certainty (or confidence) in the body of evidence for each outcome assessed.	Section 3.5, Table 5

Section and Topic	Item #	Checklist item	Location where item is reported
evidence			
**DISCUSSION**			
Discussion	23a	Provide a general interpretation of the results in the context of other evidence.	All points addressed in Discussion section
	23b	Discuss any limitations of the evidence included in the review.	
	23c	Discuss any limitations of the review processes used.	
	23d	Discuss implications of the results for practice, policy, and future research.	
**OTHER INFORMATION**			
Registration and protocol	24a	Provide registration information for the review, including register name and registration number, or state that the review was not registered.	All information for 24a-27 found from line 487 to start of references
	24b	Indicate where the review protocol can be accessed, or state that a protocol was not prepared.	
	24c	Describe and explain any amendments to information provided at registration or in the protocol.	
Support	25	Describe sources of financial or non-financial support for the review, and the role of the funders or sponsors in the review.	
Competing interests	26	Declare any competing interests of review authors.	
Availability of data, code and other materials	27	Report which of the following are publicly available and where they can be found: template data collection forms; data extracted from included studies; data used for all analyses; analytic code; any other materials used in the review.	

From: Page MJ, McKenzie JE, Bossuyt PM, Boutron I, Hoffmann TC, Mulrow CD, et al. The PRISMA 2020 statement: an updated guideline for reporting systematic reviews. BMJ 2021;372:n71. doi: 10.1136/bmj.n71

- For more information, visit: http://www.prisma-statement.org/
